# In-Shoe Foot Temperature Patterns During Lying, Sitting and Standing Postures: Baseline Data from Healthy Individuals

**DOI:** 10.3390/s26072119

**Published:** 2026-03-29

**Authors:** Stephen Mizzi, Tiziana Mifsud, Anabelle Mizzi, Mark Borg, Robert Farrugia, Owen Falzon

**Affiliations:** 1 Department of Podiatry, University of Malta, Msida MSD 2080, Malta; tiziana.mifsud.06@um.edu.mt (T.M.); anabelle.mizzi@um.edu.mt (A.M.); mborg2005@gmail.com (M.B.); robert.farrugia@um.edu.mt (R.F.); owen.falzon@um.edu.mt (O.F.); 2 Tarsos Ltd., Zabbar ZBR 1061, Malta

**Keywords:** foot thermography, in-shoe temperature, postural effects, diabetic foot, peripheral arterial disease, plantar thermal distribution

## Abstract

**Highlights:**

**What are the main findings?**

- Normative in-shoe plantar temperature patterns differ across lying, sitting, and standing, with the arch consistently warmest and the toes coolest.
- Posture significantly alters regional temperature dynamics, with uniform warming during supine acclimatization and mild cooling of distal regions during standing.

**What are the implications of the main findings?**

- Provides normative in-shoe temperature baselines in healthy individuals to support future interpretation of thermographic data in diabetes and peripheral arterial disease populations.
- Supports the use of embedded smart insoles for continuous monitoring where traditional surface thermography is not feasible.

**Abstract:**

This study aimed to establish normative in-shoe plantar foot temperature patterns across three static postures—lying, sitting, and standing—in healthy individuals, providing a clinically relevant baseline for interpreting in-shoe thermograms in diabetic or peripheral arterial disease (PAD) populations. A single-center prospective study included 20 healthy adults (40 limbs; 22–74 years) who underwent vascular and neurological screening prior to data collection. Plantar temperature was continuously recorded using Tarsos^®^ Smart Insoles with 21 embedded sensors per foot during three consecutive 10 min phases: supine, sitting, and standing. Data were analyzed for regional differences across the toes, metatarsals, arch, and heel using statistical and visual methods. Distinct posture-related temperature patterns were observed. The arch consistently exhibited the highest temperatures, while the toes remained cooler across all phases. Supine positioning resulted in relatively uniform temperature increases, whereas sitting and standing demonstrated more-stable but region-specific patterns, with slower rates of temperature change and more pronounced regional variation. Compared with barefoot thermography, the in-shoe condition showed greater heat retention and reduced evaporative cooling, highlighting the importance of context-specific baseline data. These findings demonstrate the influence of posture on plantar thermal distribution in the in-shoe environment and support the use of embedded monitoring systems for continuous assessment where surface thermography is not feasible.

## 1. Introduction

Foot temperature regulation is closely linked to vascular and metabolic processes, making it a valuable marker for assessing circulatory health and detecting early foot complications in diseases such as diabetes [1]. Advances in thermographic technology have enabled non-invasive assessment of foot temperature patterns by identifying deviations from established normative profiles. Muralidhara et al. [2] and Nagase et al. [3] demonstrated that foot thermograms can effectively distinguish between diabetic and non-diabetic individuals by analyzing temperature asymmetry and deviations from the typical “butterfly pattern” observed in healthy subjects. Similarly, Cruz-Vega et al. [4] and Khandakar et al. [5] reinforced the use of foot thermography in detecting diabetic foot complications, highlighting significant differences in plantar temperature distribution compared to healthy controls.

Establishing normative thermoregulatory baselines is clinically essential, particularly for evaluating deviations in patients with diabetes or peripheral arterial disease (PAD). Gatt et al. [6] contributed to this by providing normative thermographic data for healthy individuals, revealing symmetrical temperature distributions across contralateral limbs with specific, consistent patterns in the toes. These findings support the use of thermography as a non-invasive tool for baseline temperature assessment, offering a benchmark for identifying deviations linked to vascular insufficiency or neuropathy. Collectively, these studies emphasize the value of understanding baseline temperature distributions, as deviations from these patterns often indicate related foot-health complications.

While most thermographic studies have investigated plantar foot skin temperature under unshod supine conditions, although valuable, these conditions do not reflect real-world clinical environments where the foot remains enclosed within footwear for most of the day. Footwear alters heat transfer dynamics through insulation, reduced evaporative cooling, moisture retention, and localized pressure, fundamentally modifying plantar temperature distributions. As a result, barefoot thermographic baselines may not translate directly to useful clinical interpretation in everyday contexts. In-shoe sensing, therefore, represents an important step towards capturing physiologically and clinically meaningful thermal behavior.

Wearable systems such as smart insoles and smart textiles have been developed to monitor plantar temperature under real-world conditions. Existing studies have largely focused on device validation and activity-related temperature responses, rather than establishing controlled normative baselines under standardized conditions. For example, Najafi et al. [7] demonstrated the feasibility of smart textile systems during gait, while Billings et al. [8] evaluated temperature responses using a smart compression sock across multiple activities.

In addition to research prototypes, commercially available wearable systems for plantar temperature and pressure monitoring have been developed, building on prior work in continuous in-shoe thermometry [9,10]. These systems are primarily designed for monitoring and early detection rather than for establishing controlled normative in-shoe temperature profiles. Furthermore, they typically employ a limited number of sensing locations and do not isolate posture-dependent thermal effects.

In contrast, the present study employs a high-density in-shoe sensing system within a controlled experimental framework to characterize posture-dependent plantar temperature patterns in healthy individuals. By establishing normative in-shoe thermal profiles under defined static conditions, this work provides a preliminary baseline that complements existing wearable monitoring systems and supports improved interpretation of continuous in-shoe plantar temperature measurements.

Posture also influences physiological state and significantly affects foot temperature distribution. Research by Pereira Franco et al. [11] demonstrated that posture affects blood flow, perfusion, and thermoregulation. Standing induces gravitational stresses that reduce distal perfusion, while sitting or lying may allow more uniform circulation. These posture-dependent hemodynamic shifts can affect thermographic readings and should be considered when developing models of plantar temperature behavior.

Taken together, these factors highlight a critical gap in current thermographic models, namely, the absence of normative plantar foot temperature baselines measured under realistic conditions that account for both posture and the in-shoe microenvironment. The present study addressed this gap by establishing in-shoe plantar foot temperature baselines across three static postures: lying, sitting, and standing in healthy individuals. Temperature recordings were obtained using an insole with a dense array of embedded sensors, enabling detailed characterization of posture-dependent plantar temperature dynamics across different postures within a closed footwear environment, an aspect not captured in previous normative thermographic datasets that were obtained under barefoot conditions. By capturing the combined effects of footwear and posture on foot temperature regulation, our findings provide a more clinically relevant reference model. Identifying deviations from these patterns may enable early, non-invasive detection of conditions such as PAD or diabetic neuropathy.

## 2. Materials and Methods

Prior to commencement, this study was approved by the Research Ethics Committee of the University of Malta. Informed consent to participate in this study was obtained from all the participants. This single-center, prospective study was conducted on healthy adults without a history of significant medical, surgical, vascular, or neurological disease. Demographic data such as age, height, weight and sex were recorded for each participant.

### 2.1. Data Collection Setup

**Figure 1:**
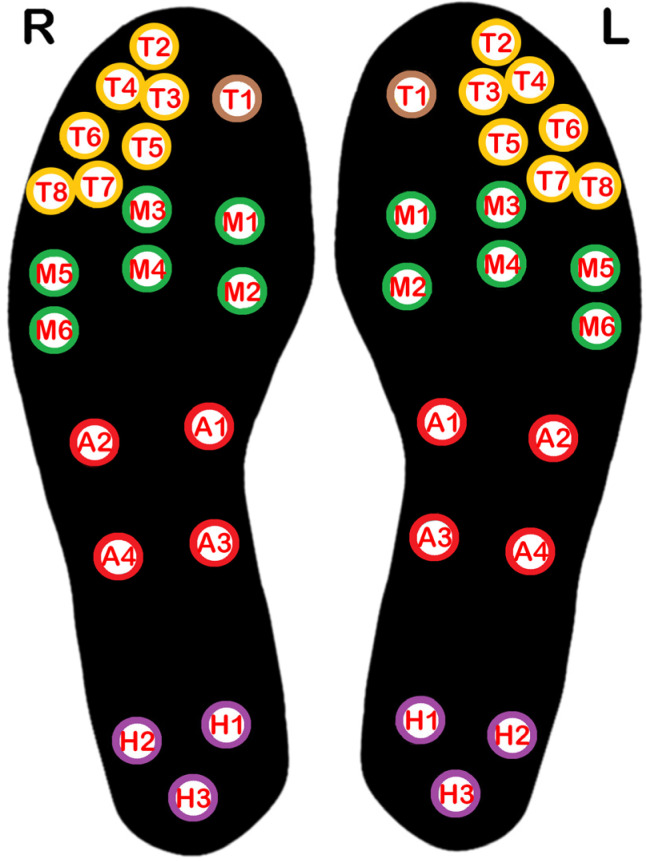
Spatial positioning of each sensor with color coding: Hallux (brown), lesser toes (yellow), metatarsal area (green), midfoot (red), and heel (purple)—Tarsos^®^ Smart Insoles.

Data collection was performed in an examination room with a controlled mean temperature of 22.5 °C, and relative humidity between 55 and 60; ambient conditions were monitored using a calibrated temperature and humidity data logger; Testo 175H1 Data logger (Testo SE & Co. KGaA, Titisee-Neustadt, Germany). The foot temperature data were acquired using Tarsos^®^ Smart Insoles (Tarsos Ltd., Zabbar, Malta) with 21 embedded temperature sensors, distributed across the insole to capture temperature from key foot regions on the plantar surface of the foot (Figure 1). These included the hallux, lesser digits, metatarsal area, arch, and heel. The insoles were connected to a mobile app via Bluetooth and recorded temperature readings at 10 s intervals.

### 2.2. Experimental Protocol

Vascular Assessment: Peripheral arterial perfusion was assessed by Doppler waveform analysis, ABPI (Ankle–Brachial Pressure Index) and TBPI (Toe–Brachial Pressure Index) by a clinician with over 10 years of experience in the field. Waveform analysis of the dorsalis pedis and posterior tibial arteries, and ABPI and TBPI measurements were taken according to published guidelines [12,13], using the Huntleigh Dopplex Assist Vascular Package^®^ (Cardiff, UK). Following standard criteria [13], waveforms were classified as triphasic (normal) or biphasic/monophasic (abnormal, indicative of PAD). Measurements were performed after a 20 min rest in a supine position. Recruited participants had triphasic waveforms, an ABPI between 1 and 1.29, and a TBPI > 0.7. Participants with a history of alcohol abuse or smoking were excluded.

Neurological Assessment: A 10 g Semmes Weinstein monofilament test was conducted on both feet at five points to screen for peripheral sensory neuropathy [14]. A 128 Hz tuning fork was used to assess for vibration perception at the dorsum of the interphalangeal joint of the hallux [15]. Only participants with normal sensation at all points were included in the study.

### 2.3. In-Shoe Temperature Data Acquisition

Data collection commenced with the vascular and neurological examinations referred to in the previous section. Subsequently, the smart insoles were inserted into the participants’ own shoes, and they were asked to put on their socks and shoes. No standardization of shoe type or lace closure was imposed. To reduce variability in the in-shoe microenvironment, participants were asked to wear standard cotton socks of similar thickness during the measurements. All data were obtained under in-shoe conditions, and recorded temperatures represent the interface between the plantar surface, sock layer, and insole sensors.

For every participant, in-shoe temperature data were collected continuously at 10 s intervals, following this structured protocol:

1. Lying Down Supine (10 min): Participants lay supine on a couch, and data were recorded for 10 min to establish in-shoe foot temperature while in a completely rested state. During this phase, the foot was allowed to acclimatize to the in-shoe microclimate.
2. Sitting (10 min): Participants sat in a seated position and in-shoe temperature data were recorded for a further 10 min. This phase established in-shoe foot temperature patterns while in a seated position.
3. Standing (10 min, stationary): Participants stood up in a stationary position while foot temperature data were recorded for a further 10 min. This phase established in-shoe foot temperature patterns of healthy individuals while in a standing position.

Data were collected from 40 limbs of 20 healthy individuals (15 males and 5 females), aged 22 to 74 years (37.12 ± 14.04). Additional demographic data, including height, weight and foot size, were recorded. The mean BMI was 27.76 ± 5.87, and the average foot size was EU 43.38 ± 2.21. Data collection involved recording temperature measurements from both the left and right feet of each participant, yielding 42 foot temperature measurements per individual. In total, 840 temperature curves were generated.

### 2.4. Data Processing and Analysis

The raw data from the Tarsos^®^ Smart Insoles, recorded in CSV format, were processed in Python (version 3.13) to produce sensor-specific plots illustrating temperature behavior over time. Data cleaning involved removing flat lines and eliminating outliers identified using a z-score threshold of ±3. Signal dropouts were addressed through temporal interpolation when gaps were minimal. Such instances represented a small proportion of the dataset and were limited to short intervals, minimizing potential influence on the estimation of temperature change rates. The cleaned temperature data were then analyzed at specific time points during each phase (t = 0 min, 2.5 min, 5 min, 7.5 min and 10 min) to compare regional differences in foot temperature. Boxplots and linear graphs were created to illustrate the temperature distribution across foot regions (toes, metatarsal area, arch, and heel) during the different static phases (lying, sitting, and standing). To produce the temperature curves per foot region, timeseries data from all participants were temporally aligned to a common start point, truncated to 10 min (if longer), and then averaged. This alignment was necessary because as participants underwent the same sequence of static phases, synchronizing the start of each phase, allowed for direct comparison of temperature behavior across these phases.

A linear mixed-effects model (LMEM) was used to assess whether the rate of temperature increase varies across foot regions. Foot region and time were included as fixed effects, with their interaction (time × foot region) to allow both baseline temperature and temperature change over time to differ across regions. Participant ID was included as a random effect, with both a random intercept and a random slope for time, to account for repeated measurements and individual differences in temporal trends. Because temperature data were collected from both feet of each participant, this modeling approach also accounts for within-subject correlation between limb measurements. Using R-style mixed-effects notation, the model structure can be expressed as temperature ~ time × foot area + (1 + time|patient), where time and foot area are modeled as fixed effects with their interaction, and participant is included as a grouping factor with both random intercepts and random slopes for time. The reference foot region was the arch.

Age and BMI were recorded for descriptive purposes but were not included as covariates in the LMEM due to the modest sample size and the primary aim of establishing overall normative plantar temperature patterns. Within each 10 min phase, temperature evolution was approximated using a linear model to estimate rates of change over time. Model fit was visually inspected and residual distributions were examined to confirm that the linear approximation was appropriate for the relatively short observation periods.

For statistical comparisons, the arch region was selected as the reference site due to its anatomical and physiological relevance in plantar perfusion. This region is supplied by the plantar arterial arch (arcus plantaris), which provides distal branches to the metatarsal heads and toes, making it central to foot vascularization [16]. Owing to its consistent perfusion and relatively stable thermal characteristics, the arch serves as a reliable and physiologically meaningful benchmark for evaluating temperature variations across other plantar regions. The rate of foot temperature change (°C/min) of each region (hallux, toes, metatarsal area and heel) was compared to the rate of foot temperature change of the arch area. Data from each foot were also analyzed separately and recorded in an Excel spreadsheet for further analysis.

## 3. Results

Across the three non-ambulatory phases, lying down (acclimatization), sitting, and standing, distinct temperature trends emerged that highlight a change in foot temperature while shod. During the acclimatization lying down supine phase, a gradual, uniform increase in temperature across all foot regions was noted. The sitting phase demonstrated more-stable temperature patterns, with a slower rate of temperature increase, and regional differences became more pronounced, while during the standing phase, a slight drop in temperature was noted across all regions except for the arch. Specifically, the arch consistently maintained the highest temperature across all phases, while the toes maintained the lowest temperature.

### 3.1. Supine Phase (10 min)

**Figure 2:**
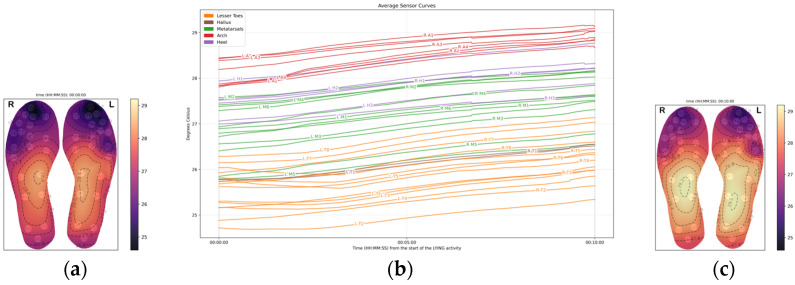
Average temperature data across the 20 participants during the lying phase: (**a**) Average foot thermogram at the start; (**b**) average temperature data curves; (**c**) average foot thermogram at the end.

Over the 10 min acclimatization period, a consistent increase in temperature was observed across all foot regions, as evidenced by the upward trends in the temperature curves shown in Figure 2. Temperature differences between observed regions were relatively stable, with similar patterns being observed at the start and throughout the supine phase. The arch exhibited the highest initial temperature during this phase, averaging 28.3 °C ± 0.3 °C, which was approximately 1 °C higher than both the metatarsals and the heel regions (27.1 °C ± 0.6 °C; 27.6 °C ± 0.3 °C, respectively), and over 2 °C higher when compared to the hallux and lesser toe regions (26.0 °C ± 0.01 °C; 25.4 °C ± 0.6 °C, respectively).

During the supine posture, the arch region showed a statistically significant temperature increase at a rate of 0.080 °C per minute (*p* < 0.001). Compared to the arch, the rate of foot temperature increase at the

- **Hallux** was slightly faster at 0.088 °C/min (*p* = 0.041);
- **Heel** was slower at 0.073 °C/min (*p* = 0.015);
- **Metatarsal area** did not differ significantly from the arch in their temperature change rates (*p* = 0.486);
- **Lesser toes** did not differ significantly from the arch in their temperature change rates (*p* = 0.120).

Overall, throughout the acclimatization phase, all regions increased by approximately 0.8 °C.

At the end of the supine phase, the arch exhibited the highest temperature, at 29.52 °C (±2.56). All other foot regions were cooler. In comparison to the arch

- The metatarsal area was approximately 1.23 °C cooler;
- The heel was 0.81 °C cooler;
- The hallux was 2.29 °C cooler;
- The lesser toes were 2.57 °C cooler.

**Figure 3:**
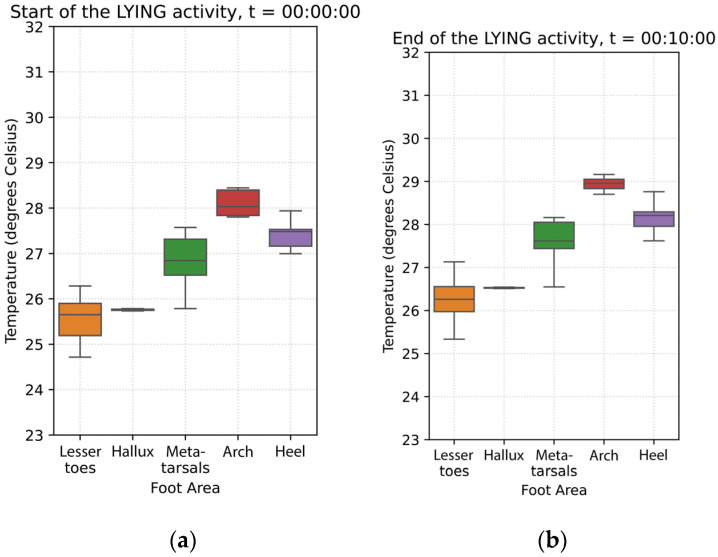
The boxplots above show the temperatures grouped by foot region at (**a**) the start of the lying phase, (**b**) at the end of the lying phase.

At the end of the ten-minute acclimatization phase, while supine and shod, all temperature differences across foot regions were highly statistically significant (*p* < 0.001). This indicates clear and meaningful variation in baseline temperatures across different foot areas, with the toes and hallux remaining substantially cooler than the arch (Figure 3).

### 3.2. Sitting Phase (10 min)

**Figure 4:**
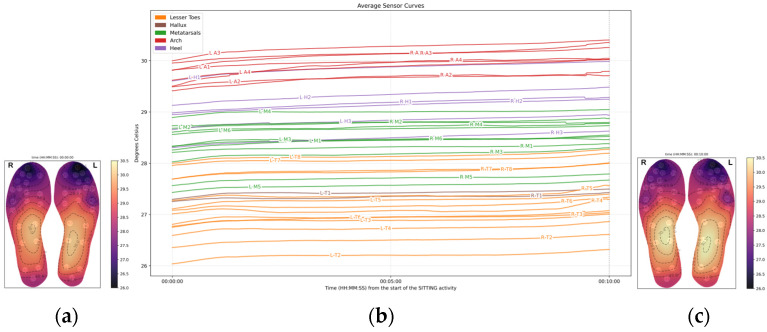
Average temperature data across the 20 participants during the sitting phase: (**a**) Average foot thermogram at the start; (**b**) average temperature data curves; (**c**) average foot thermogram at the end.

During the sitting phase, foot temperatures continued to increase in all foot regions, with a general upward trend observed from the start to the end of this phase, as shown in Figure 4. During this phase, the arch showed a statistically significant temperature increase at a rate of 0.024 °C per minute (*p* = 0.010). Compared to the arch, the rate of foot temperature increase at the

- **Metatarsals** was slower (0.018 °C/min), with a small but statistically significant difference (*p* = 0.021);
- **Heel** was slightly faster (0.034 °C/min), also showing a statistically significant difference (*p* = 0.001), though the effect size was small;
- **Hallux** and **lesser toes** (*p* = 0.4) did not differ significantly from the arch in their temperature change rates.

In summary, although the arch shows a modest but significant temperature increase during the sitting posture, temperature changes across other foot regions are broadly comparable. Statistically significant deviations for the heel and metatarsal area are small in magnitude (<0.01°C/min), suggesting that temperature dynamics after the supine acclimatization phase and during sitting are relatively uniform across the foot.

At the end of the sitting phase, the arch exhibited the highest temperature, at 29.63 °C (±2.16). All other foot regions were cooler. In comparison to the arch,

- The metatarsal area is 1.54 °C cooler;
- ⁠The heel is 0.79 °C cooler;
- The hallux is 2.61 °C cooler;
- ⁠The lesser toes are 2.80 °C cooler.

**Figure 5:**
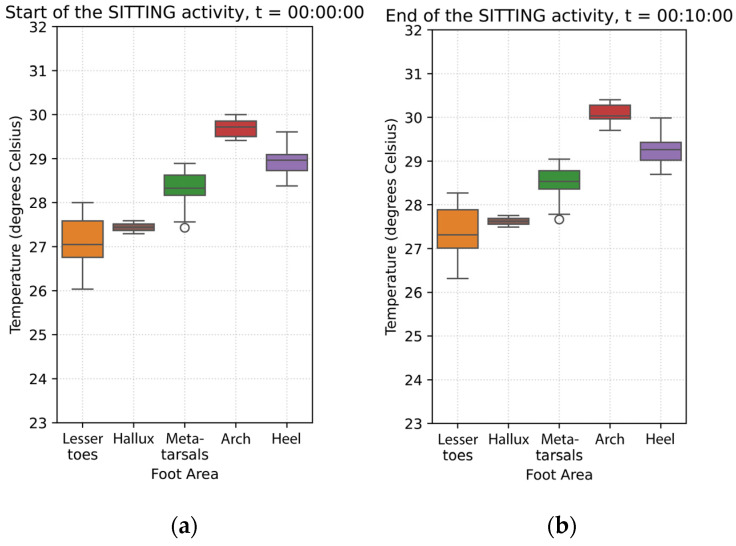
The boxplots above show the temperatures grouped by foot region at (**a**) the start of the sitting phase, (**b**) at the end of the sitting phase.

All temperature differences across all regions were statistically significant (*p* < 0.001), indicating that the hallux and toes remain markedly cooler than the arch (Figure 5).

### 3.3. Standing (10 min, Stationary)

During the standing phase, the arch exhibited a modest temperature increase at 0.02 °C/min, but this change was not statistically significant (*p* = 0.892).

Compared to the arch, the rate of foot temperature change decreased in all the regions:

- **The metatarsals** showed a decrease (−0.01 °C/min), but statistically significant (*p* < 0.001).
- **The heel** was similar to the metatarsal area, with a decrease in temperature change of −0.01 °C/min.
- **The hallux** showed a decrease in temperature (−0.03 °C/min), significantly different from the arch (*p* < 0.001), though the effect size is small.
- **The lesser toes** showed a decrease in temperature (−0.03 °C/min, *p* < 0.001), at a rate significantly faster than the rate observed in the arch.

**Figure 6:**
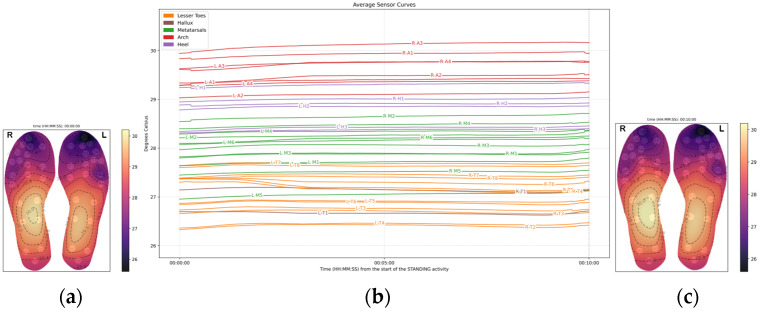
Average temperature data across the 20 participants during the standing phase: (**a**) Average foot thermogram at the start; (**b**) average temperature data curves; (**c**) average foot thermogram at the end.

These findings suggest that during standing, temperature responses across foot regions were variable, with most areas showing a cooling trend (Figure 6).

Although there was a minimal change in temperature, at the end of the standing phase, the arch still exhibited the highest temperature, at 29.63 °C (±2.68). All other foot regions were cooler in comparison:

- The metatarsal area is 1.64 °C cooler than the arch.
- The heel is 0.9 °C cooler than the arch.
- The hallux is 2.88 °C cooler than the arch.
- The lesser toes are 3.05 °C cooler than the arch.

**Figure 7:**
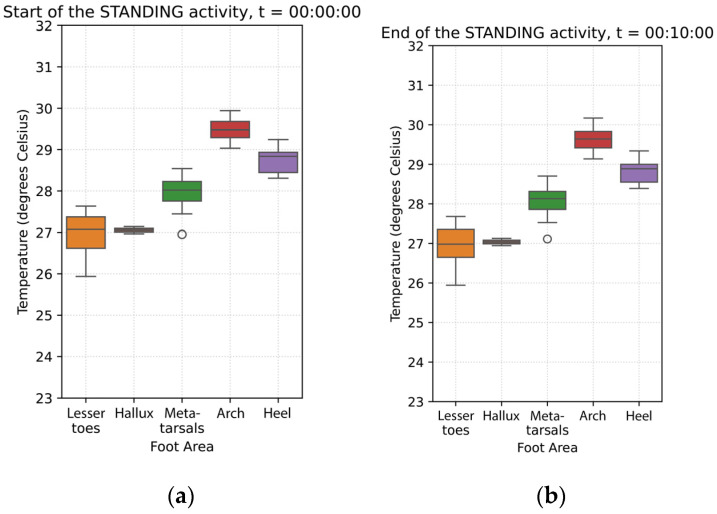
The boxplots above show the temperatures grouped by foot region at (**a**) the start of the standing phase, (**b**) at the end of the standing phase.

All regional temperature differences were statistically significant (*p* < 0.001) compared to the arch area (Figure 7).

### 3.4. Summary of Plantar Temperature Trends Across Postures

**Figure 8:**
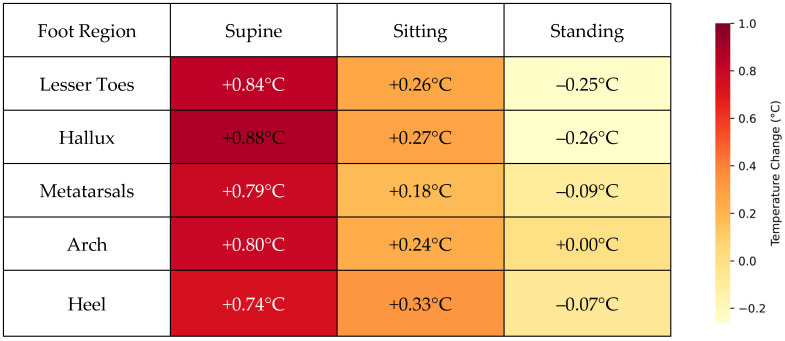
Mean temperature change (°C) across foot regions during supine, sitting, and standing postures. Participants completed three consecutive 10 min phases (supine, sitting, and standing). Values represent the mean change in temperature from the start to the end of each phase. Color intensity reflects the magnitude of temperature change across phases and regions.

The plantar temperature trends are illustrated in Figure 8, which presents a heatmap of mean temperature changes across different foot regions and postural phases. The table visually highlights a consistent increase in mean temperature during the acclimatization phase, while supine across all foot regions. This is followed by a moderate increase in temperature during the sitting phase, though the changes are less pronounced. In the standing phase, a slight decrease in temperature is observed across most regions, except for the arch, where the temperature remains stable.

## 4. Discussion

This study presents, for the first time, normative in-shoe plantar foot temperature patterns across three non-ambulatory postures, lying, sitting, and standing, in a healthy population. These findings contribute to the growing field of thermographic foot research by addressing the combined effects of posture and footwear on plantar thermal dynamics, two variables often overlooked in prior research, which focused primarily on unshod or supine conditions.

The supine posture in this study served not only as a baseline rest condition but also as an acclimatization phase, allowing the foot to adjust to the thermal characteristics of the in-shoe environment. This phase demonstrated the most uniform and substantial increase in foot temperature across all regions, particularly in the arch and heel regions. Importantly, these early thermal changes occurred entirely within a closed in-shoe system, where the insulating properties of footwear restrict airflow and promote moisture and heat retention. Unlike barefoot thermal imaging studies, the in-shoe environment imposes unique constraints on thermoregulation, emphasizing the role of footwear in shaping plantar temperature profiles. These dynamics would not be visible using surface thermography once footwear is donned. Therefore, accurate assessment of in-shoe thermal behavior requires embedded sensor-based monitoring systems capable of capturing real-world microclimate conditions, particularly relevant for populations with altered sweat or vascular responses, such as individuals with diabetes or peripheral arterial disease. These observations emphasize that the early increase in temperature dynamics observed during the acclimatization phase in the supine position should not be interpreted solely as a physiological vascular response, but rather as the result of interacting environmental factors, including metabolic heat retention, reduced evaporative cooling, and shoe–sock insulation. Consequently, clinical assessments using in-shoe temperature measurement must account for this acclimatization period to avoid overestimating perfusion when temperatures are recorded immediately after putting on shoes.

Beyond the acclimatization effects observed in the supine phase, both sitting and standing postures exerted clear influences on regional plantar temperature dynamics. The arch consistently demonstrated the highest temperature, regardless of posture, suggesting stable perfusion. In contrast, the toes, particularly the lesser toes, were consistently cooler than the arch, followed by the hallux, metatarsal and heel respectively. This pattern is consistent with the typical “butterfly pattern” observed in unshod healthy subjects as previously shown by Muralidhara et al. [2] and Nagase et al. [3]. Despite inter-individual variability in absolute temperature values, the hierarchical pattern observed across plantar regions remained consistent across participants and postures, with the arch persistently warmer and the toes cooler.

Beyond reinforcing the well-established “butterfly pattern” in plantar thermography, this study provides novel insights into inter-regional variability in the rate of temperature change across postures. During the supine acclimatization phase, temperature increases were relatively uniform across all foot regions, reflecting stable circulatory conditions and passive thermal equilibration within the in-shoe microenvironment. However, during the sitting and standing phases, posture-induced hemodynamic shifts introduced greater dispersion in thermal responses between regions.

The standing posture elicited further divergence, with all regions except for the arch exhibiting temperature reductions, likely reflecting postural vasoconstriction as a normal physiological response to prevent orthostatic hypotension [17]. Importantly, such cooling trends, though physiological in healthy individuals, may be misinterpreted as pathological (e.g., early perfusion deficits) in at-risk populations if postural context is not considered. These findings underscore the need to account for body position when interpreting in-shoe thermographic data, particularly in clinical settings where posture-dependent variations may influence diagnostic accuracy. From a clinical perspective, in diabetic foot monitoring, plantar temperature asymmetries exceeding 2.2 °C are commonly considered clinically significant and may indicate localized inflammation or early tissue injury. The normative in-shoe temperature patterns reported here may therefore assist interpretation of such thresholds during continuous plantar temperature monitoring.

Future work will involve evaluating in-shoe plantar temperature patterns in individuals with diabetes across varying levels of peripheral perfusion, enabling direct comparison with normative data presented in this study. Moreover, longitudinal intra-subject monitoring may provide greater clinical insight than cross-sectional comparisons with normative values, as deviations from an individual’s baseline temperature profile can be detected using continuous in-shoe sensing. Continuous temperature monitoring may also support predictive algorithms for ulceration risk by enabling automated detection of persistent localized hotspots or delayed thermal recovery patterns.

The relatively elevated mean BMI of the cohort (27.76 kg/m^2^) may have influenced plantar thermal dynamics, as body composition can affect peripheral perfusion and plantar pressure distribution.

Footwear characteristics, including shoe type and lace closure, were not standardized and may have influenced in-shoe thermal dynamics. Variability in footwear construction and fit represents a potential confounding factor, as differences in insulation, ventilation, and pressure can alter the in-shoe microenvironment. While footwear closure has been shown to affect plantar temperature through changes in perfusion and shear stress, particularly during walking [18], lace tightness may also influence heat exchange under static conditions by altering local pressure, perfusion, and insulation. Although dynamic loading was minimized, such variability may still have contributed to differences in measured temperature patterns. However, as the primary outcomes of this study relate to relative temperature difference, such as regional distributions and inter-limb asymmetries, the influence of footwear variability on these comparative measures is likely to be less pronounced, particularly under controlled static conditions.

Although standard cotton socks were used, the sock layer may influence heat transfer by acting as an insulating interface that attenuates or delays temperature changes. Thus, recorded values reflect the in-shoe environment rather than absolute skin temperature. As the study aimed to replicate realistic in-shoe conditions, the presence of a sock layer reflects typical use rather than an experimental artifact. While comparison with barefoot conditions (e.g., thermography) could provide a reference for true plantar skin temperature, such conditions would not adequately represent the thermal characteristics of the shod foot. As the present study is specifically concerned with temperature behavior under typical day-to-day use, including the combined effects of socks and footwear, barefoot measurements may not capture the relevant in-shoe temperature characteristics and dynamics of interest.

Although posture-dependent regional differences were described, formal statistical interaction between posture and plantar region was not explicitly modeled and may warrant investigation in larger datasets. Furthermore, while measurements were obtained under controlled static postures and dynamic gait may introduce additional thermal variations, the static conditions examined here provide a clinically relevant baseline for interpreting in-shoe non-ambulatory plantar temperature patterns.

## 5. Conclusions

These normative profiles provide a vital reference for interpreting foot thermograms in diabetic or peripheral arterial disease (PAD) populations. The identification of typical temperature hierarchies (arch > heel ≈ metatarsals > hallux > lesser toes) within a realistic in-shoe environment offers a baseline for detecting anomalies such as asymmetry, delayed warming, or abnormal cooling. Furthermore, our data support integrating smart insole technologies in continuous-monitoring frameworks, enabling real-time detection of temperature deviations indicative of local ischemia, pressure injury, or autonomic dysregulation.

## Data Availability

The data presented in this study are available on request from the corresponding author due to institutional and ethical considerations, as the dataset contains information that could potentially compromise participant privacy.

## Abbreviations

The following abbreviations are used in this manuscript:

PAD	Peripheral Artery Disease
ABPI	Ankle–Brachial Pressure Index
TBPI	Toe–Brachial Pressure Index
LMEM	Linear Mixed-Effects Model
